# A Conserved Role for LRRK2 and Roco Proteins in the Regulation of Mitochondrial Activity

**DOI:** 10.3389/fcell.2021.734554

**Published:** 2021-09-08

**Authors:** Katharina E. Rosenbusch, Asmaa Oun, Oana Sanislav, Sui T. Lay, Ineke Keizer-Gunnink, Sarah J. Annesley, Paul R. Fisher, Amalia M. Dolga, Arjan Kortholt

**Affiliations:** ^1^Department of Cell Biochemistry, University of Groningen, Groningen, Netherlands; ^2^Groningen Research Institute of Pharmacy (GRIP), Molecular Pharmacology XB10, Groningen, Netherlands; ^3^Department of Biotechnology, Institute of Graduate Studies and Research, Alexandria University, Alexandria, Egypt; ^4^Department of Physiology Anatomy and Microbiology, La Trobe University, Melbourne, VIC, Australia; ^5^Department of Pharmacology, Faculty of Medicine, Suleyman Demirel University, Isparta, Turkey

**Keywords:** Parkinsion’s disease, LRRK2, Roco protein, *Dictyostelium discoideum*, mitochondria

## Abstract

Parkinson’s Disease (PD) is the second most common neurodegenerative disease world-wide. Mutations in the multidomain protein Leucine Rich Repeat Kinase 2 (LRRK2) are the most frequent cause of hereditary PD. Furthermore, recent data suggest that independent of mutations, increased kinase activity of LRRK2 plays an essential role in PD pathogenesis. Isolated mitochondria of tissue samples from PD patients carrying LRRK2 mutations display a significant impairment of mitochondrial function. However, due to the complexity of the mitochondrial signaling network, the role of LRRK2 in mitochondrial metabolism is still not well understood. Previously we have shown that *D. discoideum* Roco4 is a suitable model to study the activation mechanism of LRRK2 *in vivo*. To get more insight in the LRRK2 pathways regulating mitochondrial activity we used this Roco4 model system in combination with murine RAW macrophages. Here we show that both *Dictyostelium roco4* knockout and cells expressing PD-mutants show behavioral and developmental phenotypes that are characteristic for mitochondrial impairment. Mitochondrial activity measured by Seahorse technology revealed that the basal respiration of *D. discoideum roco4-* cells is significantly increased compared to the WT strain, while the basal and maximal respiration values of cells overexpressing Roco4 are reduced compared to the WT strain. Consistently, LRRK2 KO RAW 264.7 cells exhibit higher maximal mitochondrial respiration activity compared to the LRRK2 parental RAW264.7 cells. Measurement on isolated mitochondria from LRRK2 KO and parental RAW 264.7 cells revealed no difference in activity compared to the parental cells. Furthermore, neither *D. discoideum roco4-* nor LRRK2 KO RAW 264.7 showed a difference in either the number or the morphology of mitochondria compared to their respective parental strains. This suggests that the observed effects on the mitochondrial respiratory in cells are indirect and that LRRK2/Roco proteins most likely require other cytosolic cofactors to elicit mitochondrial effects.

## Introduction

Neurological disorders pose an increasing societal and economic burden within our aging population. Parkinson’s Disease (PD) is the second most common neurological disorder affecting 1–2% of the population over 65 years old (Eichinger et al., 2005; Li et al., 2015). PD is characterized by the progressive loss of dopaminergic neurons in the substantia nigra pars compacta and the formation of fibrillar protein aggregates enriched in α-synuclein proteins, called Lewy bodies (Parkinson, 1817; Dauer and Przedborski, 2003). The majority of PD cases are sporadic or idiopathic whereas an increasing amount of familial forms of PD are being identified (Paisán-Ruíz et al., 2004; Zimprich et al., 2004; Karimi-Moghadam et al., 2018). PD-associated mutations have been found in 23 genes or loci, including *SNCA* (α-synuclein), *PARK2* (parkin), *PINK1*, *PARK7* (DJ-1) and *LRRK2* (leucine-rich repeat kinase 2) (Healy et al., 2008). Mutations in *LRRK2* genes cause an autosomal dominant hereditary type of PD and accounts for 5% of the cases among European and North American PD patients (Li et al., 2015). Importantly, it was shown recently that LRRK2 kinase activity was enhanced in post-mortem brain tissue from patients with idiopathic PD (iPD), suggesting that independent of mutations, wild-type LRRK2 plays a role in PD pathology as well (Di Maio et al., 2018).

LRRK2, a large multi-domain protein with a size of 286 kDa, belongs to the Roco protein family (Marín et al., 2008; van Egmond and van Haastert, 2010; Guaitoli et al., 2016). It consists of an N-terminal Armadillo domain, Ankyrin repeats, a LRR (leucine rich repeats) domain, a Roc (Ras of complex protein domain), a COR (C-terminal of Roc) domain, a kinase and a C-terminal WD40 domain (Mata et al., 2006; van Egmond and van Haastert, 2010; Guaitoli et al., 2016). Interestingly, the LRRK2 protein possesses two distinct enzymatic functions; a GTPase activity of the Roc and a kinase activity associated with the kinase domain (Mata et al., 2006; Guaitoli et al., 2016). Several mutations distributed over the *LRRK2* gene are associated with PD, with the G2019S mutation located within the kinase domain, being the most common mutation (Ozelius et al., 2006; Li et al., 2015). On the molecular level, it has been shown that all common LRRK2 PD mutations cause increased LRRK2 kinase activity and/or decreased GTPase activity (West et al., 2005, 2007; Greggio et al., 2006; Jaleel et al., 2007; Luzón-Toro et al., 2007; Aasly et al., 2010; Sheng et al., 2012; Ho et al., 2016). However, the underlying pathways involved in LRRK2- associated signaling with PD pathology are still not completely understood. LRRK2 has been linked to a wide range of biological processes and several LRRK2 kinase substrates have been proposed (Boon et al., 2014; Wallings et al., 2015; Roosen and Cookson, 2016; Rosenbusch and Kortholt, 2016; Tang, 2016). LRRK2 signaling is involved in mitochondrial regulation, autophagy and cell death, actin and microtubule dynamics, synthesis and transport of vesicles as well as neurotransmitter, immune responses, and the activity of the intestinal network (Esteves et al., 2014).

Impaired mitochondrial activity is a common characteristic of both, iPD and familial PD, and several genes linked to PD play an important role in mitochondrial quality control (Narendra et al., 2008; Youle and van der Bliek, 2012; Liu et al., 2017; Toyofuku et al., 2019; Delcambre et al., 2020; Weindel et al., 2020). Even though the mitochondrial morphology was found to be affected e.g., in age mutant Parkinsonian LRRK2(R1441G) mice, mitochondrial shape does not qualify as a biomarker as it is mainly detected in late stages of PD and/or post mortem and inconsistent with the disease pattern (Liu et al., 2020). One theory regarding the underlying trigger of PD involves the presence and accumulation of oxidative stress. Samples of the substantia nigra of PD patients have been found to exhibit increased levels of oxidized DNA, proteins, lipids, and overall ROS levels (Bender et al., 2006; Knoefler et al., 2013; Verma et al., 2014). Further experiments in yeast showed that over-expression of LRRK2 and hence its enzymatic activities triggered the loss of the mitochondrial transmembrane potential, ATP depletion, decreased respiratory capacities, generation of ROS and finally cell death (Aufschnaiter et al., 2018). Consistently, immortalized lymphocytes from individuals with iPD display dramatic elevations of mitochondrial respiratory activities (Annesley et al., 2016). The first indication that LRRK2 plays a role in mitochondrial signaling came from the observations that LRRK2 is localized to mitochondria in transfected HEK-293T cells and in rat brain models (West et al., 2005; Biskup et al., 2006). Subsequently, it was shown that overexpression of WT and PD-associated mutants of LRRK2 in SH- SY5Y cell line and in primary cortical neurons resulted in impaired mitochondrial fission and consequently the accumulation of fragmented mitochondria (Knoefler et al., 2013). This effect on mitochondrial dysfunction is dependent on LRRK2 enzymatic activity and the presence of the fission protein DLP1 (dynamin-like protein 1) (Niu et al., 2012; Wang et al., 2012; Su and Qi, 2013). Moreover, enhanced levels of fragmented mitochondria have also been found in PD patient-derived fibroblast lines with the LRRK2 G2019S mutation (Mortiboys et al., 2010; Smith et al., 2016). Higher levels of mitochondrial fission, CD68 (active microglia maker), Drp1 (mitochondrial fission marker) as well as a shortage of microglial processes were also found in the striatum brain area of LRRK2 G2019S transgenic mice (Ho et al., 2018). Addition of LPS (lipopolysaccharide) triggered enhanced mitochondrial fission and Drp1 levels, an effect which was rescued via the addition of the LRRK2 kinase inhibitor GSK2578215A (Ho et al., 2018). Also the PD associated LRRK2 mutation E193K displayed abnormal scaffold protein activity via an increased binding to Drp1 in primary fibroblasts and thus enhancing mitochondrial fission levels (Carrion et al., 2018). Fibroblasts from LRRK2 mutation G2019S carrier patients in turn demonstrated enhanced autophagy in response to mitochondrial challenging condition, indicating that the exhaustion of mitochondrial bioenergetics and autophagic reverse might contribute to the development of PD phenotypes (Juárez-Flores et al., 2018). Furthermore, brain samples isolated post-mortem from human G2019S PD patients showed increased phosphorylation of peroxiredoxin 3 (PRDX3), antioxidant of thioredoxin peroxidase family, and reduced endogenous peroxidase activity and increased oxidative damage (Angeles et al., 2011). A recent study suggests that LRRK2 also regulates mitochondrial quality control by modulating ER–mitochondrial tethering by modulating the PERK-dependent ubiquitination pathway under ER stress conditions (Toyofuku et al., 2019). The G2019S mutation induced dissociation of LRRK2 from the E3 ubiquitin ligases MARCH5, MULAN, and Parkin, allowing PERK to phosphorylate and activate these enzymes, resulting in the ubiquitination of their mitochondrial substrates.

From these data it is clear that LRRK2 plays a role in mitochondrial control and/or signaling. However the exact effect LRRK2 mutations have on mitochondrial bioenergetics remains to be identified. Here we used the amoebic model organism *Dictyostelium discoideum* (*D. discoideum*) to further study the effect of Roco/LRRK2 mutations on mitochondrial morphology and activity. *D. discoideum* possesses 11 Roco proteins and has been successfully investigated with regards to gaining insight in the complex activation mechanism of LRRK2/Roco proteins and mitochondrial disease (Barth et al., 2007; van Egmond and van Haastert, 2010; Francione et al., 2011; Gilsbach et al., 2012). Previously, we have shown that Roco4 plays an important role in the late development stage of *D. discoideum* (van Egmond and van Haastert, 2010). Upon starvation cells lacking *roco4* initially undergo the characteristic developmental phases, however, after 12 h of starvation the cells start to display severe developmental defects (see also below). The formation of slugs and subsequent stalks and spore heads is severely delayed and *roco4* knockout (*roco4-*) cells display aberrant fruiting body morphology as the spore heads are located on the agar surface due to instable stalks. Interestingly, replacement of the Roco4 kinase domain with the LRRK2 kinase domain results in the restoration of functional Roco4 protein *in vivo* (Gilsbach et al., 2012). Furthermore, the LRRK2 mutation G2019S is conserved in Roco4, and corresponds to the G1179S mutation resulting likewise in an increased kinase activity (Gilsbach et al., 2012). Here, we showed that both *roco4-* and G1179S-expressing *D. discoideum* cells show behavioral and developmental phenotypes that are characteristic for mitochondrial impairment. Both mutant cell lines show a severe development phenotype and atypical spore morphology. The G1179S strain displayed lower mitochondrial activity, while *roco4-* strain displayed higher mitochondrial activity and ROS production compared to the WT strain although mitochondrial morphology was normal. We have verified these results for LRRK2 in murine macrophages RAW 264.7 cells, highly expressing endogenous LRRK2, and LRRK2 knockout (KO) RAW 264.7 cells. Together, our data show that *D. discoideum* Roco4 and human LRRK2 have a conserved role in the regulation of mitochondrial activity.

## Results

### Altered *D. discoideum* Roco Protein Activity Results in Mitochondrial Disease-Like Phenotypes *in vivo*

**FIGURE 1:**
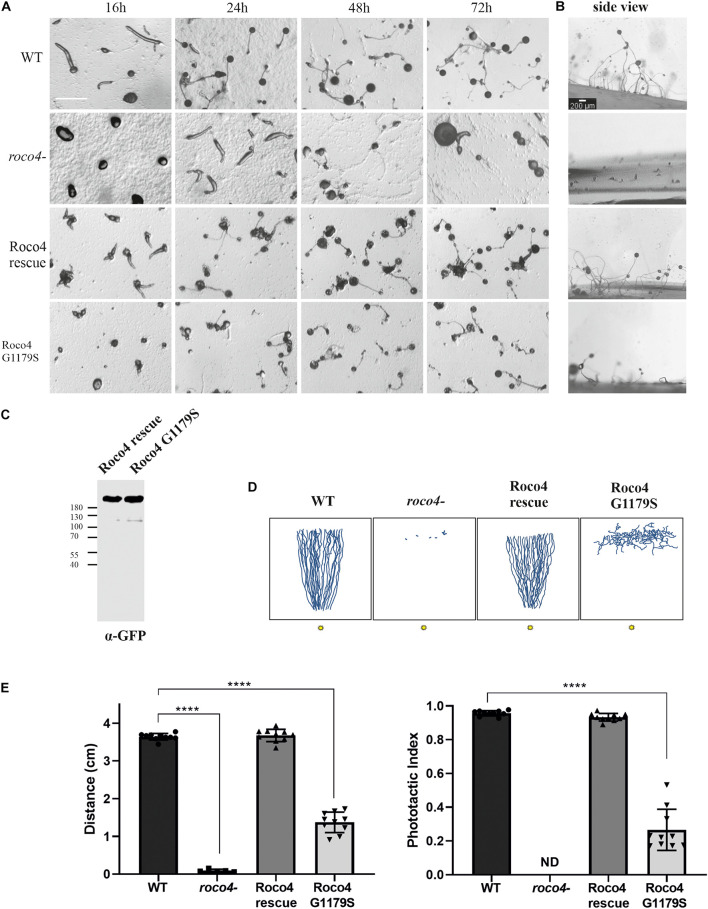
Roco4 mutants have impaired development and phototaxis. **(A)** WT, *roco4*-, Roco4 rescue and Roco4 G1179S cells were plated on NN agar plates and images were taken at the indicated time points. Scale bar indicates 200 μm. **(B)** Side view of fruiting bodies after the completion of the developmental cycle. Scale bar indicates 200 μm. **(C)** Western blot of lysate of cells expressing Roco4-GFP and Roco4 G1179S-GFP cells. **(D)** Representative illustration of slug tracks. The 3 cm wide trench where cells started is positioned horizontally at the top and the 1 mm wide light slit is located at the bottom of the image. Illustrations of the scanned slug tracks have been made via ImageJ. **(E)** Phototactic properties of the indicated strains. Shown are the total distance moved (left panel) and the phototactic index (ratio of the total distance moved and the direct distance moved to the light. The data shown are the means of 20–25 tracks. **** indicates *P* < 0.005 differences from WT in an ANOVA test.

*D. discoideum* possesses two different life cycles: a vegetative cycle, responsible for cell division, and a developmental cycle initiates upon nutrient depletion that culminates in the formation of dormant spores. During the developmental cycle *D. discoideum* cells aggregate to form a multicellular mobile organism, called a slug, which is able to move in the direction of light and heat, a process called photo-and thermotaxis, respectively, till it settles down to form the spore stalk and head. Roco4 expression is elevated during the later slug phase (> 12 h) and consistently, *roco4-* cells have previously been found to display severe development phenotypes, including delayed development and abnormal spore morphology (Figures 1A–E; van Egmond and van Haastert, 2010). The amino acid that is most frequently mutated in PD (G2019S) is conserved in Roco4 and corresponds to G1179S. Previously we have shown that both LRRK2G2019S and Roco4G1179S showed increased kinase activity *in vitro* (Kortholt et al., 2012). Interestingly, expression of Roco4 G1179S *in vivo*, results in a similar phenotype compared to *roco4-* cells (Figures 1A–C). Wild-type (WT) cells start forming slugs after 12 h, whereas both *roco4-* and Roco4 G1179S cells still remain in the mound stage after 12 h (Figure 1A). The two mutant cell lines remain in the mound stage for an excessive time period followed by multiple attempts to culminate. They eventually generate slugs and form fruiting bodies only after > 50 h (Roco4 G1179S) and > 70 h (*roco4-*) of starvation, respectively (Figure 1A). Furthermore, *roco4-* cells hardly make stalks, while Roco4 G1179S make much smaller stalks compared to WT (Figure 1B). Consistent with previous studies, we found that re-expression of the Roco4 WT protein (Roco4 rescue) completely restored the developmental defects (Figures 1A–C; van Egmond and van Haastert, 2010; Kortholt et al., 2012).

We next addressed whether the impaired development might be accompanied by a defect in phototaxis. Therefore, cells were washed, placed and developed on a non-nutrient agar (NNA) plate with a given light source in front. After 3 days the tracks of the migrated slug were blotted onto a plastic sheet, stained with Coomassie dye and the trails were quantified (Figures 1D,E). Wild-type cells showed highly efficient phototaxis toward the light source, with a phototactic index of 0.96 and a track length of 3.64 cm. In contrast both *roco4-* and G1179S cells showed severely impaired phototaxis. The *roco4-* slugs hardly move (Supplementary Movie 1) and therefore the accuracy of directional movement is indeterminate. The G1179S slugs do displace although impaired (1.37 cm) and with very poor directional movement toward the light (Figure 1E).

Together our results show that both the absence of Roco4 as well as the hyperactivity of the Roco4 kinase lead to impaired *D. discoideum* development and phototaxis. Interestingly, delayed development, impaired stalk formation and dysregulated phototaxis have been strongly linked to impaired mitochondrial function (Francione et al., 2011).

### *D. discoideum* Roco Mutant Cells Have Enhanced ROS Production

**FIGURE 2:**
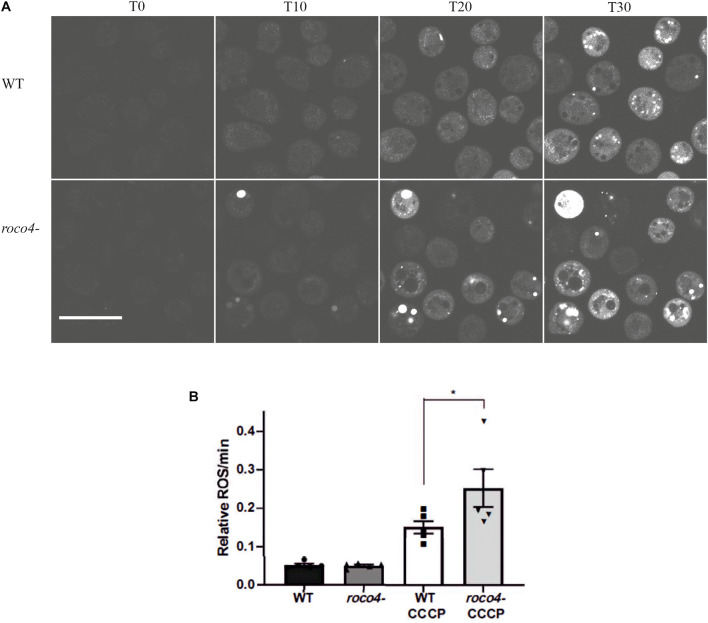
ROS production in *D. discoideum* cells. **(A,B)** Six hours starved WT and *roco4-* cells were incubated with DCF-DA. **(A)** After addition of CCCP images were taken at the indicated time points (“T” minutes). Scale bar indicates 20 μm. **(B)** Total levels of ROS production were determined by measuring the intensity of the DCF signal using a microplate reader (Synergy HTX). Results are displayed as relative values to each other based on the increasing signal intensity. Shown are the means of 5 independent experiments, * indicates *P* < 0.05 difference from the WT control in a paired students *t*-test.

To analyze the mitochondrial activity in the Roco4 mutants we determined the accumulation of reactive oxygen species (ROS) levels by analyzing the dye intensity of 2′,7′-Dichlorofluorescin diacetate (DCF-DA). This dye is colorless until it gets oxidized and generates the fluorescent product 2′,7′-dichlorofluorescein (DCF) (Brubacher and Bols, 2001). Accumulation of DCF signal thus gives an indication of the amount of redox-active substances produced in the cell. WT and *roco4*- cells were washed with phosphate buffer to limit background noise and subsequently DCF-DA was added in the presence and absence of the Carbonyl cyanide m-chlorophenylhydrazone (CCCP). Mitochondrial uncoupler, CCCP, induces depolarization of the mitochondrial membrane and triggers maximum mitochondrial activity. The fluorescent signal accompanying the oxidation of DCF-DA to DCF has been measured after addition of CCCP. Initially WT and *roco4*- cells have similar low levels of ROS (Figure 2A), however, following CCCP addition the ROS production in *roco4-* cells appears to increase more compared to WT. Interestingly, in several *roco4-* cells the DCF fluorescence was so high that the complete cell is fluorescent and no individual patches can be observed (Figure 2A). To quantitatively analyze the DCF fluorescence intensity we used a plate reader assay (Figure 2B) and the vegetative WT and mutant cells were measured over time in the presence or absence of CCCP (Figure 2B). Consistent with the microscopy data, both cell lines display a similarly low level of initial ROS concentrations and *roco4-* cells show a significant increase of ROS levels following 30 min of CCCP challenge (Figure 2B).

### Roco Proteins and LRRK2 Modulate Mitochondrial Respiration

**FIGURE 3:**
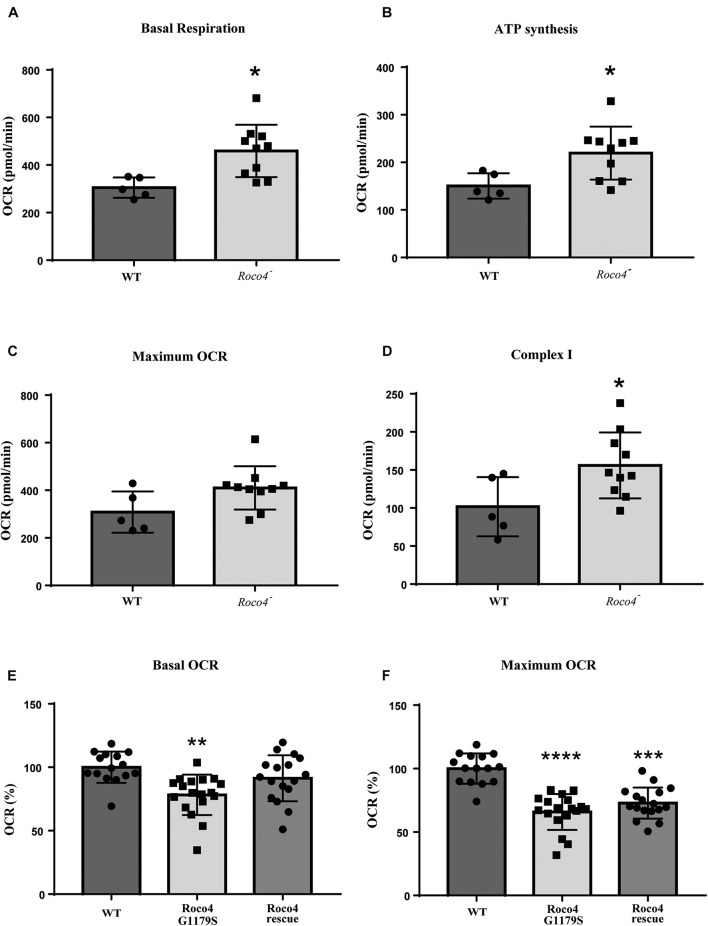
Mitochondrial respirometry measurement in *D. discoideum* cells. The *roco4-* strain has enhanced mitochondrial respiratory activity while the Roco4 G1179S shows decreased mitochondrial respiration compared to the WT strain. Oxygen Consumption Rate (OCR) measurements were obtained over time (min) using an extracellular flux analyzer (Seahorse Bioscience). The mitochondrial stress test was used to obtain bioenergetic parameters. The figure shows the OCR values of the *D. discoideum* strains. **(A–D)** The *roco4-* strain has significantly elevated basal OCR, ATP synthesis rate and complex I activity compared to the WT. **(C)** The maximum OCR is slightly elevated though not significant. **(E,F)** Reduction in the basal and maximum OCR of the Roco4 G1179S strain relative to the WT while the Roco4 rescue cells show reduction only in the maximum OCR compared to the WT cells. All data are represented as mean ± SD. Statistical significance was assessed using unpaired *t*-test for the WT and the *roco4-* strains and one-way ANOVA with Tukey’s post-test or Kruskal–Wallis test for the WT, Roco4 G1179S and Roco4 rescue strains. *P*-values indicating statistically significant differences between the mean values compared to the WT control and are defined as follows: *p < 0.05, **p < 0.01, ***p < 0.001, and ****p < 0.0001.

To determine if the observed increase in ROS production is due to altered mitochondrial functioning, we analyzed the mitochondrial respiration of the WT, *roco4*-, Roco4 G1179S, and Roco4 rescue strains using the Seahorse Bioscience XF24 Extracellular Flux Analyzer (Figure 3). Seahorse technique enables kinetic measurement of oxidative metabolism by measuring the rate of oxygen consumption [oxygen consumption rate (OCR)] in real time. This method can provide detailed information on the metabolic phenotype by the addition of mitochondrial substrates and modulators of mitochondrial electron transport chain (Annesley et al., 2016). Cells were added to the Seahorse analyzer and basal OCR levels were determined prior to port injections and represent energetic demand of the cell under baseline conditions (Figures 3A,E). The decrease in OCR following the injection of ATP synthase inhibitor (DCCD or oligomycin) indicates the ATP synthesis rate (Figure 3B) while the maximum OCR is attained by adding the uncoupler (CCCP or FCCP) (Figures 3C,F). Complex I activity can be calculated by the decrease in OCR after rotenone addition (complex I inhibitor) (Figure 3D). Our data revealed that the basal mitochondrial respiration, ATP synthesis rate and complex I activity in the *roco4-* strain are significantly increased, while the maximum OCR is slightly increased yet not statistically significant compared to the WT strain (Figures 3A–D). In contrast, Roco4 G1179S and the Roco4 rescue cells exhibit a significantly reduced maximal respiration compared to the WT strain, while Roco4 G1179S cells have significantly lower basal respiration as well (Figures 3E,F). These data indicate that *D. discoideum* Roco4 mutants have an impact on different steps in the mitochondrial respiratory activity.

**FIGURE 4:**
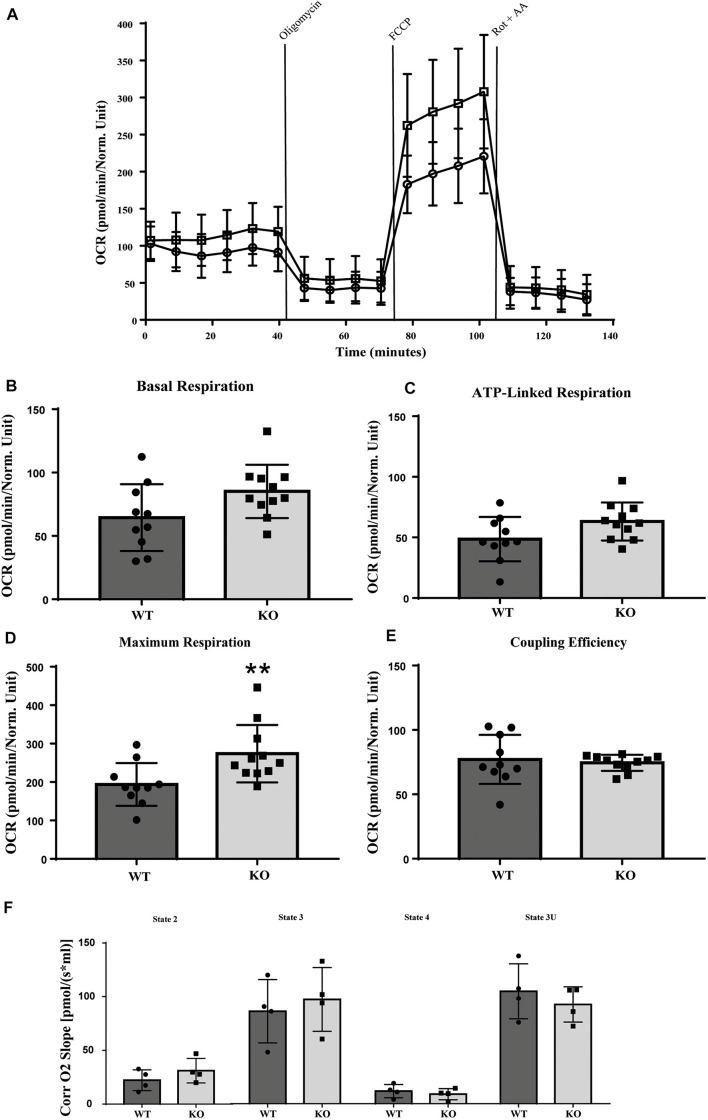
Mitochondrial respirometry measurement in RAW 264.7 cells. LRRK2 KO RAW 264.7 cells have significantly higher maximum OCR in intact cells compared to LRRK2 parental RAW 264.7 cells while in isolated mitochondria the mitochondrial respiration is not altered. **(A)** Line graph of the OCR values of LRRK2 parental (closed circle) and KO RAW 264.7 (open square) cells in a representative seahorse measurement in intact cells. **(B–E)** Bar graphs showing different mitochondrial respiratory parameters for LRRK2 parental (black bar) and KO RAW 264.7 (gray bar) derived from the seahorse analysis. Data are represented as mean ± SD and statistical significance was computed using unpaired *t*-test or Mann-Whitney test. Data shown are representative of 10- 11 different wells/condition and the experiment was repeated at least three times with individual LRRK2 parental RAW 264.7 and LRRK2 KO RAW 264.7 macrophage cells. *P*-values indicating statistically significant differences between the mean values compared to the WT control with **p < 0.01. **(F)** Corrected oxygen slope of different mitochondrial respiration states of LRRK2 parental (black bar) and KO RAW 264.7 (gray bar) cells revealing no significant differences in the mitochondrial respiration states in isolated mitochondrial fraction. Four independent experiments were performed and 3–4 measurements/cell line were measured in each experiment. Data are shown as mean ± SD of the average of four experiments.

LRRK2 is highly expressed in immune cells and several studies have linked LRRK2 signaling to inflammation (Thévenet et al., 2011; Brockmann et al., 2017; Dzamko, 2017; Lee et al., 2017). We therefore used murine LRRK2 parental and LRRK2 KO RAW 264.7 macrophages to analyze whether the absence of LRRK2 (Knocking out) also has an effect on mitochondrial activity. LRRK2 KO RAW 264.7 cells show a higher maximal respiration and increased spare capacity compared to LRRK2 parental RAW 264.7 cells, whereas basal respiration, ATP-production and coupling efficiency [(ATP production/basal respiration)^∗^100] were not significantly altered (Figures 4A–E). Previous studies have shown that the majority of LRRK2 is cytosolic and, if at all, only a small fraction of LRRK2 is present in mitochondria (West et al., 2005; Papkovskaia et al., 2012). We used high-resolution respirometry (Oroboros) to measure the activity of isolated mitochondria. Mitochondrial respiration was measured in the presence of the mitochondrial substrates, pyruvate, and malate (state 2). ADP was added in a saturating concentration to determine phosphorylating respiration (state3). Subsequently, oligomycin was applied to inhibit ATP synthase (state 4) and FCCP provided the maximal uncoupled-stimulated respiration (state 3U). Consistent with the proposed cytosolic LRRK2 localization we did not observe any significant differences in any of the mitochondrial respiration states between LRRK2 parental and KO RAW 264.7 cells, and can conclude that the OXPHOS complexes and complex V are functionally normal (Figure 4F).

Together our data show that *D. discoideum* Roco4 and LRRK2 mutants affect the mitochondrial respiration activity in a similar way. Furthermore, the differences between the whole cell and mitochondrial fraction measurements, suggest that this effect is indirect and that LRRK2 most likely requires other cytosolic cofactors to elicit mitochondrial effects.

### Roco Mutants Do Not Affect Either the Number or the Morphology of Mitochondria

**FIGURE 5:**
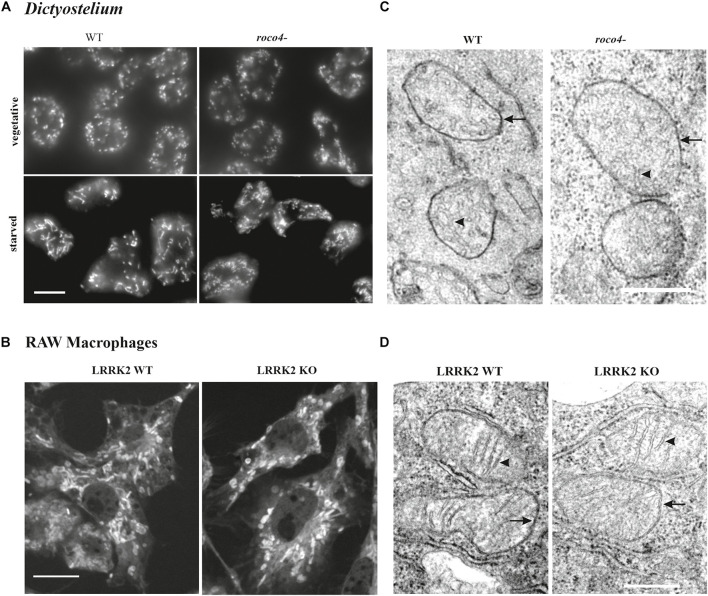
Disruption of Roco4 or LRRK2 does not affect the number and morphology of mitochondria. **(A)** *D. discoideum* WT and *roco4-* cells in vegetative state and after starvation, stained with Alexa-594 conjugated streptavidin. Scale bar indicates 10 μm. **(B)** Macrophages, LRRK2 parental and LRRK2-KO strains, stained with MitoTracker^TM^ Deep Red. Scale bar indicates 10 μm. **(C,D)** TEM images of mitochondria in the respective *D. discoideum* **(C)** and macrophage **(D)** cells. The mitochondrial inner and outer membrane layers are indicated with arrows whereas cristae with arrowheads. Scale bars indicate 1 μm.

To gain information on the shape and number of mitochondria in the *D. discoideum* cells, we used a specific mitochondrial marker coupled to Alexa-594-conjugated streptavidin (Life Technologies). Alexa-594-streptavidin binds to the heavily biotinylated protein 3-methylcrotonyl-CoA carboxylase α, which is localized in the mitochondrial lumen (Davidson et al., 2013). WT and *roco4-* cells show a similar mitochondrial network both in vegetative cells and in starved cells. In both strains mitochondrial elongation is enhanced upon starvation due to increasing energy demands (Figure 5A; Katz, 2002). The fixed cells show very clear elongated and interconnected mitochondria with no visible fragmentation in either WT *or roco4-* cells. Similar observations have been made in living cells using the previously published marker fluorescent marker GFP-gemA (Supplementary Figure 1; Vlahou et al., 2011; Davidson et al., 2013). Furthermore, we observed no obvious differences in mitochondrial morphology in LRRK2 parental and KO RAW 264.7 cells (Figure 5B). The ratio (percentage) of mitochondria to overall cell size was calculated using area measurements on images with ImageJ software; no differences were observed for both the *roco4-* and LRRK2 KO RAW 264.7 strains compared to the respective parental strains (Table 1). Consistently, manual counting of the mitochondria of the *D. discoideum* cells did not reveal significant differences between WT and *roco4-* cells (Table 1). The amount of mitochondria are in accordance with the previous defined number of 50 mitochondria per cell and observer focal lane in healthy WT cells (Gilson et al., 2003).

**TABLE 1:** Quantification of mitochondria and cristae mitochondria.

Strain	Area mitochondria (% of cell)	Mitochondria (number/cell)	Cristae (number/mitochondrion)
*D. d.* WT	13.7 ± 1.0	49 ± 2.0	n.d.
*D. d. roco4-*	13.9 ± 1.1	47 ± 1.5	n.d.
LRRK2 parental	12.6 ± 0.6	n.d.	5.3 ± 0.7
LRRK2 KO	11.8 ± 1.4	n.d.	5.0 ± 0.6

*D. discoideum cells were stained with 50 ng/mL Alexa-594 conjugated streptavidin (Life Technologies) for 30 min at 21°C, human RAW 264.7 cells were stained with 200 nM MitoTracker^TM^ Deep Red FM (Invitrogen) for 30 min at 37°C. The number of mitochondria of one focal plane was manually counted in fixed vegetative D. discoideum cells. Shown are the mean and SEM of 25 cells per strain. The surface area [in percent (%)] indicates the ratio of the number of pixels covering the mitochondria with regards to the number of pixels of the overall cell area in the confocal plane. Values are the mean and SEM obtained from 25 cells per strain. The cristae were manually counted from EM pictures of fixed human RAW 264.7 cells of one focal plane. The counted numbers are the mean and SEM obtained from 25 mitochondria per strain. n.d., not determined.*

To visualize qualitative ultrastructural details of the mitochondria within cells, we made use of Electron Microscopy (EM). Due to the high resolution it is possible to visualize the overall mitochondrial shape and organization and also details of the inner lumen with regards to size, folding, and organization of the cristae (Figures 5C,D and Table 1). *D. discoideum* WT and *roco4-* and RAW 264.7 WT and KO cells were fixed and visualized under the EM. The mitochondria of both *D. discoideum* cell lines are neither aggregated nor clustered in close proximity to a specific organelle (Figure 5C). Shape, size and connectivity of the mitochondria are in agreement with previous literature analyzing wild-type-like mitochondrial membranes (Arnoult et al., 2001). In accordance, EM images of LRRK2 parental and KO RAW 264.7 cells do not indicate an abnormal distribution of the mitochondria within the cell or in clusters around an identifiable organelle (Figure 5D). The mitochondria appear similar in size, shape, and connectivity compared to the overall cell volume in both cell lines (Figure 5D and Table 1). The cristae are clearly visible and display proper folding and organization as normal for mitochondria in eukaryotic cells. The differences in mitochondrial physiology as observed between parental and Roco mutants are thus not expressed in their (ultra)structural organization.

## Discussion

Although numerous studies have implicated mitochondrial dysfunction in LRRK2 pathogenesis of PD, the role of LRRK2 in mitochondrial bioenergetics is still a central unsettled query (Lin and Beal, 2006). Here we investigated the effect of mutated Roco proteins on mitochondrial functioning in an *in vivo* model system. First of all, we showed that *D. discoideum* cells lacking Roco4 and cells expressing the conserved PD mutation G1179S display strong phenotypes associated with mitochondrial malfunction: a delayed development, affected phototaxis and impaired stalk formation. Secondly, cells lacking Roco4 showed increased ROS production upon mitochondrial stress compared to the WT strain. Thirdly, using the well-established Seahorse XF Technology we showed that Roco4 mutants have altered mitochondrial bioenergetics. Under normal conditions, the cell functions using only a part of its bioenergetic capability, called basal respiration. Upon stress, the demand for extra ATP production increases (Desler et al., 2012). Our data show that *roco4-* cells display overall enhanced mitochondrial respiration compared to WT which is especially evident in the level of basal respiration, ATP synthesis and complex I activity. In contrast, the G1179S mutants cells displayed decreased mitochondrial respiration activities, making the PD-mutation more vulnerable to stress. Consistently, we found that LRRK2 KO RAW 264.7 cells exhibit significant enhanced mitochondrial respiration compared to the parental strain, especially with regards to the maximal respiration as well as spare capacities. So far contradicting effects on mitochondrial activity have been reported for LRRK2 PD mutant strains. Consistent with the Roco4 G1179S data, a previous study reported decreased basal OCRs for iPSC-derived neural cells from individuals carrying the LRRK2 G2019S and R1441C mutations compared to healthy control iPSC-derived neural cells, although the maximum OCR, ATP-synthesis and proton leak were not statistical significant changed (Cooper et al., 2012). Contrarily, the basal and maximal OCRs of fibroblasts from G2019S PD-patients and G2019S unaffected individuals exhibited comparable rates to the control fibroblasts, whereas the proton leak was increased in G2019S carrier fibroblasts independent of the disease status (Grünewald et al., 2013). Furthermore, immortalized PD lymphocytes showed enhanced mitochondrial activities as assessed via Seahorse XF measurements (Annesley et al., 2016), and ROS production was found to be enhanced in fibroblast cells from LRRK2 G2019S patient samples with higher level of oxygen damage (Yakhine-diop et al., 2014; Aufschnaiter et al., 2018; Juárez-Flores et al., 2018). Our data show that LRRK2 KO cells have increased mitochondrial maximum respiration, while LRRK2 mutations exhibit decreased maximum respiration, suggesting that alteration in LRRK2 activity in PD cells leads to impaired mitochondrial activity, however, the exact cellular effect might differ per cell strain and experimental conditions.

Interestingly, our high-resolution respirometry measurements on isolated mitochondria from LRRK2 KO and parental RAW 264.7 cells revealed no difference in activity compared to the parental cells. Furthermore, neither *D. discoideum roco4-* nor LRRK2 KO RAW 264.7 showed a difference in either the number or the morphology of mitochondria compared to their respective parental strains. This suggests that the observed effects on the mitochondrial respiratory in cells is indirect and that LRRK2/Roco proteins most likely require other cytosolic cofactors to elicit mitochondrial effects. The first evidence that LRRK2 plays a direct role in mitochondrial signaling has come from studies that reported a mitochondrial localization of LRRK2 (West et al., 2005; Biskup et al., 2006), however, others could not reproduce these findings (Greggio et al., 2007). The main reason for this discrepancy is the lack of antibodies and/or markers that convincingly visualize endogenous LRRK2 in cells. Interestingly, a more recent study that used a poly-ADP-ribose assisted protein localization assay (PARAPLAY), that overcomes the need of conventional immunocytochemistry showed that overexpressed LRRK2 does not localize to the mitochondrial matrix (Osuagwu et al., 2019). To confirm these findings for endogenous LRRK2 and understand where and how LRRK2 activation takes place it will be important to develop high-resolution LRRK2 imaging probes and/or GFP-LRRK2 knock-in models. Taken together we conclude that Roco mutant proteins lead to altered mitochondrial respiration *in vivo*, and that this effect is probably indirect. However, the levels of severity vary between the various detection methods, and different cell lines and PD models used.

In this study, we have used the *in vivo* model *D. discoideum* and RAW 264.7 macrophages. LRRK2 is highly expressed in both monocytes and microglia, the residential immune cells or the macrophage cells of the brain, and mutations of LRRK2 also underlie susceptibility to immune diseases, including leprosy and Crohn’s disease, highlighting a potential immunologic function. Furthermore, rodent microglia and peripheral blood mononuclear cells display increased expression of LRRK2 and elevated inflammatory response to endotoxin lipopolysaccharide (LPS) and interferon γ (IFN-γ), respectively (Gardet et al., 2010; Moehle et al., 2012). α-synuclein aggregation, a hallmark of PD pathology has also been shown to increase the expression of LRRK2 proteins (Lin et al., 2009). Moreover, *LRRK2*-null rodent microglia have less inflammatory processes and cytokine release in response to the LPS stimulus (Daher et al., 2014). Together these results show that LRRK2 expression and activity are critical in order to achieve a (neuro)inflammatory response. Our data on the RAW 264.7 macrophages show that LRRK2 affects mitochondrial activity. To confirm and further investigate the role of LRRK2 in regulating mitochondrial respiration in immune cells, it will be important to perform high-resolution mitochondrial respirometry measurements in disease-relevant human immune cells, including primary human monocytes and iPSC-derived microglia from PD patients https://pubmed.ncbi.nlm.nih.gov/33540322/.

## Materials and Methods

### Generation of *D. discoideum* Cell Lines

The *D. discoideum* cells were cultured in HL5 (Formedium) media. For starvation the medium was washed away and the cells were incubated in phosphate buffer (PB; 10 mM phosphate buffer, pH 6.5) for 6 h prior to the experiment. Roco4 mutants have been created and described previously. The generation of the *roco4-* cell line (DBS0350095) and the Roco4 constructs (DDB_G0288251)/G1179S (DBS0350100) has been described previously (van Egmond and van Haastert, 2010; Gilsbach et al., 2012). Transformation of the cells has been carried out according to standard protocol and the cells were cultured in the presence of the according selection marker (van Egmond and van Haastert, 2010). Expression of the constructs was verified via Western Blot. For this, vegetative cells were washed in PB and lysed in 1:2 volume ratio in SDS buffer (200 mM Tris, pH 6.8, 400 mM DTT, 8% SDS, 0.05 Bromophenol Blue, 20% Glycerol) at 95°C for 10 min. Cell lysate was loaded on a SDS-PAGE gel, subsequently blotted on a nitrocellulose membrane and incubated with α-GFP according to the standard procedure (Gilsbach et al., 2012) [“GFP Antibody (B-2)” and “m-IgGκ BP-HRP,” Santa Cruz Biotechnology].

### Origin and Cultivation of Macrophages

The murine cells were obtained from ATCC: parental RAW 264.7 and the LRRK2-KO (knockout) RAW 264.7. The cells were cultured in DMEM (LGC Standards GMBH) supplemented with 10% fetal bovine serum and 1% penicillin–streptomycin at 37°C in 5% CO_2_.

### Phenotypic Assays of *D. discoideum*

Starvation and onset of the developmental cycle have been induced by washing *D. discoideum* cells with PB. 2 × 10^7^ cells were spread on *ϕ*5 cm 1.5% non-nutrient agar. Developmental time and stalk formation was monitored and documented using a Stemi SV 11 (Zeiss) microscope. For phototaxis, 5 × 10^5^ cells were washed, resuspended in 10 μL and placed in a trench that was cut out on the far end of an agar plate. The plate was placed in a dark *ϕ*6 cm tube with a 1 mm wide open light slit opposite the cells. Cells were allowed to absolve the developmental cycle (24–72 h). Slugs were formed that migrated toward the light source over the agar surface. Slug tracks, consisting of slime and cell debris were blotted to a PVC sheet, stained with Coomassie Blue and scanned via a CanonScan 9000F (Canon). The tracks were digitized and measured via ImageJ and the phototaxis index is expressed as the ratio of the absolute distance traveled to the light divided by the total track length.

### Visualization of Mitochondria

For mitochondrial staining, *D. discoideum* cells were settled on glass plates, then fixed with ultracold (–80°C) Ethanol, washed with PB and incubated with 50 ng/mL Alexa-594 conjugated streptavidin (Life Technologies) for 30 min with no subsequent washing steps according to protocol (Davidson et al., 2013). Cell images were taken via a Zeiss Axio Observer microscope. The number of mitochondria was manually counted in 25 cells per cell line. The percentage area of the cell occupied by mitochondria was calculated using ImageJ as the ratio of mitochondrial area to the whole cell area.

Mitochondria in the macrophage cell lines were stained with a mitotracker dye: cells (250,000 cells/well) were grown overnight on coverslips. Then the growth medium was replaced with a fresh medium containing 200 nM MitoTracker^TM^ Deep Red FM (Invitrogen) and incubated for 30 min at 37°C, followed by washing twice with PBS and fixation with 4% PFA for 25 min. After washing with PBS the cover slips were mounted on glass slides using Floroshield^TM^ with DAPI (Sigma) as a mounting medium. To study mitochondrial morphology, images were taken via Zeiss LSM800 confocal microscope and analyzed with ImageJ. The percentage area of the cell occupied by mitochondria was calculated as described above.

For transmission electron microscopy (TEM), *D. discoideum* and macrophage cells were grown to a confluent monolayer on Thermanox coverslips (Thermo Fisher Scientific). The coverslips with cells were fixed by immersion in ice-cold glutaraldehyde (6% in 0.1 M cacodylate-buffer pH 7.2) overnight, washed thoroughly with water and subsequently fixed and contrasted with 1% OsO_4_ in 0.1 M cacodylate-buffer pH 7.2 (1 h at RT). For *D. discoideum* KMnO_4_ (1.5% in double distilled water) was used as additional fixative. After thorough washing with water, the cells were incubated overnight in 1% uranyl-acetate. The coverslips were dehydrated in a rising ethanol series and embedded in Epon with the following steps: infiltration with increasing ratio of Epoxy-resin in ethanol (1:1, 3:1, pure resin, 15 min each step, pure resin overnight). The coverslips were cut into smaller pieces and transferred to beem capsules and covered with fresh resin which was polymerized at 60^o^C for 48 h. Ultrathin sections were cut and the samples were examined with a transmission electron microscope (Philips CM12) at 80 kV.

### Cellular ROS

For the detection of intracellular ROS levels, 5 × 10^5^ vegetative *D. discoideum* cells were settled on glass plates for 30 min. A 10 μM 2′,7′-Dichlorofluorescin diacetate (DCF-DA, Sigma-Aldrich) was added to the cells and allowed to incubate for 30 min. Images (1 frame/min) of cells have been taken with a Zeiss LSM800 microscope with and without the addition of 1 μM Carbonyl cyanide m-chlorophenylhydrazone (CCCP; Sigma- Aldrich) over a time period of 30 min. Alternatively, 5 × 10^5^ vegetative WT and mutant cells, washed and resuspended in PB, were seeded into wells of a black 96-wells-plate. A 10 μM DCF-DA was added to the wells and left to incubate for 30 min. Measurements (every minute) were taken immediately after the addition of 1 μM CCCP to the respective wells for 30 min via the microplate reader Synergy HTX (BioTek). Background measurements were taken of HL5 media with 10 μM DCF-DA and subtracted from the data. Measurements have been conducted five times.

### Seahorse Measurements

Oxygen consumption measurements were performed on intact *D. discoideum* cells using the Seahorse XFe24 Flux Analyzer (Seahorse Biosciences) as previously described (Lay et al., 2016). Seahorse measurements were performed in two groups; one for the WT and *roco4-* strains and the other for the WT, Roco4 G1179S and Roco4 rescue. Briefly, *D. discoideum* cells were collected, washed and resuspended in SIH assay medium (SIH0102, ForMedium^TM^, United Kingdom) supplemented with 20 mM sodium pyruvate and 5 mM sodium malate (pH 7.4). To ensure cell adherence during the assay, cells were seeded in XF24 cell culture plates coated with Matrigel^®^ (growth factor reduced basement membrane matrix; Corning, MA, United States) at 1 × 10^5^ cells/well and allowed to settle for 1–2 h. Bioenergetic profiling was performed by monitoring basal oxygen consumption rate (OCR) for 48 min followed by the sequential injection of the following inhibitors; N,N’-dicyclohexylcarbodiimide (DCCD; 10 μM; Sigma- Aldrich; Port A), carbonyl cyanide 3-chlorophenylhydrazone (CCCP; 10 μM; Sigma- Aldrich; Port B), rotenone (20 μM; Sigma- Aldrich; Port C), and antimycin A (10 μM; Sigma- Aldrich; Port D) at 32 min interval. Mitochondrial responses were monitored every 8 min following a cycle of 3 min mixing/2 min delay/3 min measurement. The following respiratory parameters were utilized to analyze the bioenergetic function: basal OCR, ATP synthesis rate (with DCCD), maximum OCR (with CCCP), complex I activity (with rotenone) and mitochondrial spare capacity (with antimycin A).

For bioenergetics assessment of the effect of LRRK2 on murine macrophage cells, adherent LRRK2 parental RAW 264.7 and LRRK2 KO RAW 264.7 cells were used. Cells were transferred to XF microplates at a seeding density of 75,000 cells/well 24 h before OCR measurement. After replacing the growth medium with 180 μL of assay medium supplemented with 25 mM glucose, 2 mM L-glutamine and 1 mM sodium pyruvate (pH 7.35), cells were preincubated for 1 h in a CO_2_ free incubator at 37°C before starting the assay procedure. Following basal respiration measurement, various compounds such as: oligomycin (2.5 μM; Sigma-Aldrich), Carbonyl cyanide-4-(trifluoromethoxy)phenylhydrazone (FCCP; 2.5 μM; Sigma-Aldrich), rotenone (1 μM) and antimycin A (1 μM) were sequentially injected to assess mitochondrial coupling of respiratory chain, maximal and non-mitochondrial oxygen consumption. OCR values were normalized to protein content in each well, determined with a BCA assay.

### High-Resolution Respirometry

The function of the respiratory chain on isolated mitochondria in LRRK2 parental and KO RAW 264.7 cells was assessed via high resolution respirometry at 37°C, Oxygraph 2K (Oroboros systems, Innsbruck, Austria). Mitochondrial isolation was performed using a pump-controlled cell rupture system as formerly described (Schmitt et al., 2013). Isolated mitochondria (200 μg) were injected to an air tight chamber containing Mir05 (0.5 mM EGTA, 3 mM MgCl_2_, 60 mM lactobionic acid, 20 mM taurine, 10 mM KH_2_PO_4_, 20 mM HEPES, 110 mM D-Sucrose and 1 mg/mL fatty acid-free BSA). Sequential addition of the following substances were done to measure mitochondrial respiration states; pyruvate (5 mM) and malate (2 mM) to assess non-phosphorylating respiration (mitochondrial state 2), saturating concentration of ADP (500 μM) to quantify the OXPHOS capacity of complex I-linked respiration (state 3), oligomycin (2 μg/mL) to inhibit the ATP-synthase (state 4), and stepwise titration of FCCP (1 μM) to calculate the maximal respiration (state 3U). Oxygen flux were recorded in real-time using the DatLab software and the data was analyzed with DatLab5 (version 5.1.1.91).

## Data Availability Statement

The original contributions presented in the study are included in the article/Supplementary Material, further inquiries can be directed to the corresponding author/s.

## Author Contributions

KR, PF, SA, AD, and AK designed the experimental research. KR and IK-G characterized the amoebic mutants and their mitochondrial phenotype. KR, AO, OS, and SL performed mitochondrial measurements. All contributed to writing the manuscript. All authors contributed to the article and approved the submitted version.

## Conflict of Interest

The authors declare that the research was conducted in the absence of any commercial or financial relationships that could be construed as a potential conflict of interest.

## Publisher’s Note

All claims expressed in this article are solely those of the authors and do not necessarily represent those of their affiliated organizations, or those of the publisher, the editors and the reviewers. Any product that may be evaluated in this article, or claim that may be made by its manufacturer, is not guaranteed or endorsed by the publisher.
