# Supplementary material for: A Conserved Role for LRRK2 and Roco Proteins in the Regulation of Mitochondrial Activity
Source: Front Cell Dev Biol. 2021 Sep 8;9:734554. doi: 10.3389/fcell.2021.734554 (PMC8455996; doi:10.3389/fcell.2021.734554)
Supplement: Supplementary file 2 [file Data_Sheet_1.docx]

Supplementary Material

# Supplementary Figures

**
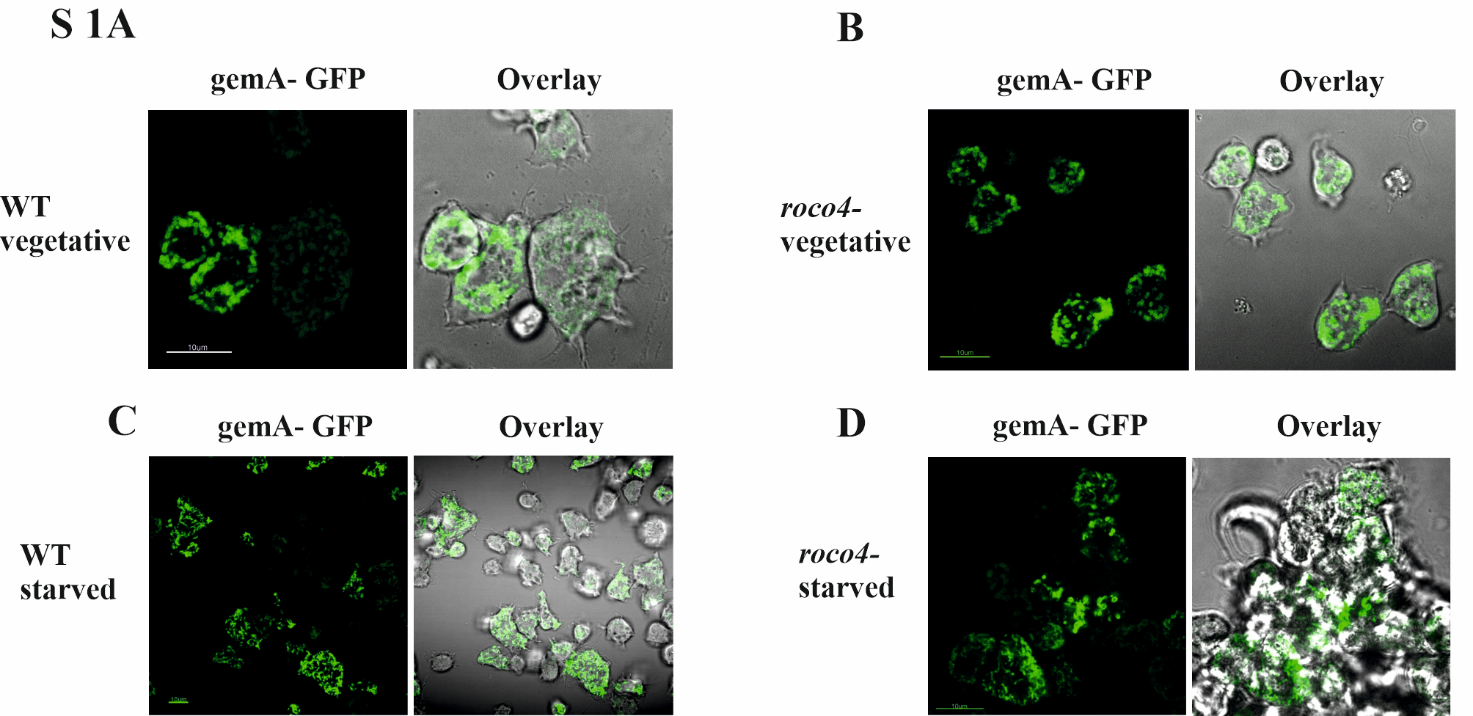
**

**Figure S1**: Visualization of mitochondria in living *Dictyostelium* cells. Representative confocal images of vegetative (A + B) and 6h starved (C+ D) WT and *roco4-* cells expressing the mitochondrial GFP-gemA marker. Inserted bar indicates 10µm.

**Supplementary Movie 1**

Vegetative *roco4-* cells were washed, resuspended in PB and approximately 2x10^7^ cells were seeded onto 5cm 1.5% non-nutrient agar plates. The movie displays the movement of *roco4-* slugs between 8h and 14 h after the onset of development. Development was monitored and documented using a Stemi SV 11 (Zeiss) microscope with a frame rate of 1/min and the movie was decimated by 3.
